# A Human-Centered Approach to CV Care: Infrastructure Development in Uganda

**DOI:** 10.1016/j.gheart.2018.02.002

**Published:** 2018-04-21

**Authors:** Christopher T. Longenecker, Ankur Kalra, Emmy Okello, Peter Lwabi, John O. Omagino, Cissy Kityo, Moses R. Kamya, Allison R. Webel, Daniel I. Simon, Robert A. Salata, Marco A. Costa

**Affiliations:** * Division of Cardiovascular Medicine, University Hospitals Cleveland Medical Center, Cleveland, OH, USA; † Department of Medicine, Case Western Reserve University School of Medicine, Cleveland, OH, USA; ‡ Uganda Heart Institute, Kampala, Uganda; § Joint Clinical Research Centre, Kampala, Uganda; ∥ Department of Medicine, Makerere University School of Medicine, Mulago Hill, Kampala, Uganda; ¶ Frances Payne Bolton School of Nursing, Case Western Reserve University, Cleveland, OH, USA.

## Abstract

In this case study, we describe an ongoing approach to develop sustainable acute and chronic cardiovascular care infrastructure in Uganda that involves patient and provider participation. Leveraging strong infrastructure for HIV/AIDS care delivery, University Hospitals Harrington Heart and Vascular Institute and Case Western Reserve University have partnered with U.S. and Ugandan collaborators to improve cardiovascular capabilities. The collaboration has solicited innovative solutions from patients and providers focusing on education and advanced training, penicillin supply, diagnostic strategy (e.g., hand-held ultrasound), maternal health, and community awareness. Key outcomes of this approach have been the completion of formal training of the first interventional cardiologists and heart failure specialists in the country, establishment of 4 integrated regional centers of excellence in rheumatic heart disease care with a national rheumatic heart disease registry, a penicillin distribution and adherence support program focused on retention in care, access to imaging technology, and in-country capabilities to treat advanced rheumatic heart valve disease.

The growing global burden of noncommunicable disease (NCD) has been well-documented [1] and is straining health systems in low-income countries that already have a high burden of infectious diseases, including human immunodeficiency virus/acquired immunodeficiency syndrome (HIV/AIDS) [2]. In sub-Saharan Africa, public and private investment in health care infrastructure is needed to translate recent successes in HIV/AIDS care to those with other chronic diseases such as cardiovascular disease and to advance human health more holistically. Yet, financial investment alone is not the cure. We believe that a human-centered, participatory approach grounded in a clear understanding of the human needs of patients, providers, and society will lead to scalable and cost-effective innovations in low-income countries [3].

In this paper, we describe a case study of cardiovascular care infrastructure development in the resource-limited East African nation of Uganda, led by university hospitals (UH) Harrington Heart and Vascular Institute and Case Western Reserve University (CWRU) School of Medicine in collaboration with key U.S. and Ugandan partners. Our overarching goal is to develop capabilities and improve heart care by focusing on sustainability and decentralization of services from its capital Kampala to other regions of the country. Our approach leverages longstanding academic collaboration and pre-existing HIV/ AIDS infrastructure and has proven to be an effective model for North-South collaboration in health sector development.

## GAINING INSIGHT

### A history of relationship building

CWRU established the first research and medical education program in Uganda in 1989, when then Chair of Medicine and Nobel Laureate Dr. Fred Robbins was invited by the government of Uganda to help respond to the HIV/AIDS epidemic. For over 25 years, CWRU and UH physicians have contributed significantly to capacity-building efforts with key Ugandan partners such as Makerere University and a network of HIV/AIDS clinics known as the Joint Clinical Research Centre (JCRC). With technical assistance from UH/CWRU, the JCRC has established a world-class infrastructure throughout Uganda to promote community awareness of HIV/AIDS, perform clinical and epidemiological research, establish treatment protocols, and train health care professionals in HIV/AIDS care [4-7].

In 2011, the UH/CWRU—Uganda collaboration expanded beyond HIV and infectious diseases to cardiovascular disease with initial support from the National Institutes of Health and Fogarty International Center through the Medical Education Partnership Initiative Cardiovascular Linked Award (R24 TW008861). The Uganda Heart Institute (UHI)—a semi-autonomous heart center at Mulago Hospital that had been building pediatric congenital heart disease surgery capacity [8] in partnership with Children’s National Medical Center (Washington, DC, USA)—was engaged early on as a key partner. In August 2012, UH cardiologists were invited through the Medical Education Partnership Initiative Cardiovascular Linked Award program to perform some of the first procedures in a new catheterization laboratory at UHI, including the first percutaneous balloon mitral valvuloplasty for rheumatic mitral stenosis [9], the first complex, multivessel percutaneous coronary intervention, and the first peripheral artery angioplasty.

A key insight from this historic visit was that the model of individual trips by external experts to treat a limited number of patients—a strategy often used throughout sub- Saharan Africa—would not effectively build capabilities and was not sustainable. Therefore, we sought a more comprehensive strategy to develop sustainable cardiovascular care capabilities in the country. The first step was to identify a single disease model that was meaningful to people living in Uganda and find a solution that would be desirable by local stakeholders and feasible to implement.

The human cost of rheumatic heart disease (RHD) affecting young adults who would otherwise contribute to society is well known [10]. Once a major cause of chronic illness in the United States, RHD has become a neglected disease in the West that nonetheless continues to ravage poor nations around the globe [11]. For many reasons, RHD was an ideal starting point for heart care expansion in Uganda because it affects both children and adults, it has a broad and deep socioeconomic impact, is diagnosed clinically with the assistance of basic cardiovascular technologies such as echocardiography, requires chronic care coordination and medication adherence, and in advanced stages, requires percutaneous or surgical intervention. The idea was simple—improving RHD care would likely improve many aspects of heart disease care in Uganda.

Our decision to focus on RHD was also timely, as the Medtronic Foundation and the World Heart Federation were considering new initiatives to eradicate RHD. In February 2013, UH/CWRU received a 5-year, $1.3 million grant from the Medtronic Foundation to “leverage longterm partnerships and existing HIV/AIDS infrastructure to create a comprehensive RHD treatment program in Uganda” [12]. In 2014, the Medtronic Foundation launched RHD Action, a $6 million commitment to a global movement of RHD technical assistance and advocacy partners, and Uganda was invited to be 1 of the 2 founding “country partners.” The collaboration was rebranded as RHD Action–Uganda. Since then, additional sources of funding and partnerships have been added (Tables 1 and 2) to widen its scope and sustainability.

### The stakeholders: Patients and providers

Rheumatic fever is an abnormal immune response to invasive group A streptococcal infection (typically pharyngitis) that primarily affects children ages 5 to 15, leading to chronic valvular RHD over time and causing heart failure and early mortality, most commonly in early adulthood [10].Penicillin is a proven treatment for rheumatic fever and chronic RHD that substantially reduces the occurrence of future attacks and progression of valve disease [13]. To be effective, however, penicillin must be administered as an injection every 4 weeks, and therefore, requires effective chronic care delivery systems [10].

Similar to RHD, patients with other cardiovascular diseases such as cardiomyopathy or congenital heart disease require chronic longitudinal care. Yet, the Ugandan health care system has limited capacity to monitor patients with chronic illnesses longitudinally [14,15]. The public health response to HIV/AIDS began to change this paradigm. As a result of the President’s Emergency Plan to Fund AIDS Relief and other programs, antiretroviral therapy is now widely available and chronic care is delivered efficiently in local clinics; however, it is not clear that the President’s Emergency Plan to Fund AIDS Relief has improved care for chronic NCDs, and in some cases may have siphoned off resources [5,16]

To improve delivery of chronic cardiovascular care services in Uganda, a human-centered participatory approach that involved both patients and providers was needed [17]. Listening to Ugandan providers, it was clear that improving the quality of cardiovascular care in Uganda would require a massive effort to train and repurpose a health care workforce. The problem as our team saw it was that Ugandans living with HIV were able to find effective and efficient care delivered by an extensive network of clinics across the country, but patients with cardiovascular diseases were left waiting in crowded clinics for hours to see clinicians who did not have the training or tools to appropriately manage the disease.

## FOSTERING INNOVATION

### A latticework on which to build

The HIV/AIDS infrastructure that had been scaled up with massive global funding was a model for quality chronic care delivery, but could it also be “leveraged” to provide quality care for other NCD services [18-20]? This question has been framed in 2 ways: (1) Can HIV/AIDS infrastructure be used to provide care for HIV-uninfected individuals with NCD? (2) Can NCD services be integrated into HIV clinics to improve the control of NCD comorbidities among people living with HIV [19]? We felt that the second approach would have the greatest impact on public health.

**FIGURE 1.**
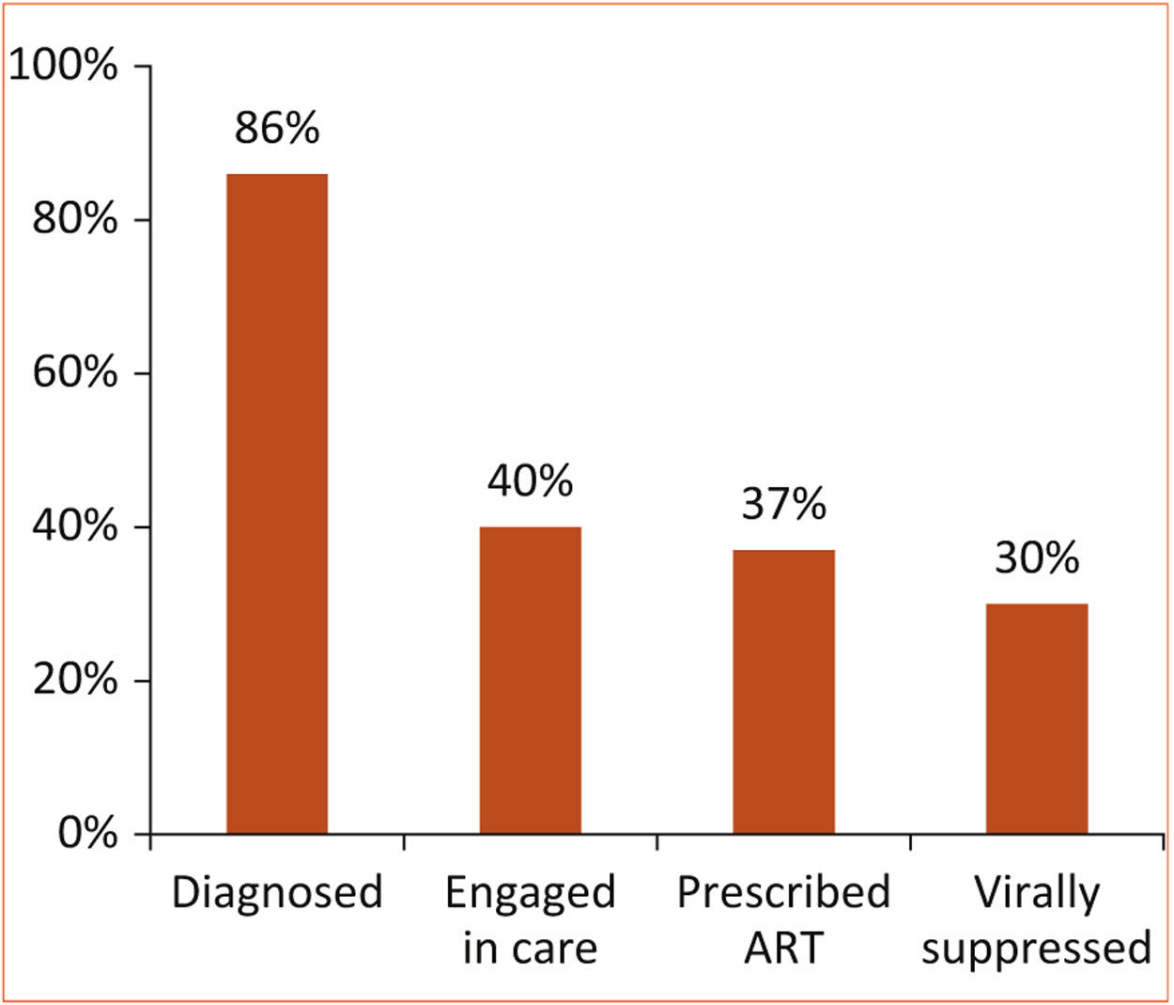
The HIV/AIDS treatment cascade in the United States. Of the approximately 1 million people living with HIV in the United States, only 30% have achieved viral suppression. Adapted from U.S. Department of Health and Human Services [22] Source data from the Centers for Disease Control and Prevention National HIV Surveillance System and Medical Monitoring Project (USA); 2011. AIDS, acquired immunodeficiency syndrome; HIV, human immunodeficiency virus.

In our case, the JCRC had already established a network of Regional Centers of Excellence for HIV care that served as nodes within a lattice network covering the entire country of Uganda. In initial brainstorming sessions and planning meetings, administrative infrastructure was the most readily identified strength to be repurposed, but additional ideas included: (1) using nurses who were trained in HIV care to deliver penicillin injections; (2) using HIV counselors to improve retention in care and adherence to penicillin; (3) using the HIV Treatment Cascade model [21,22] (Figure 1) to evaluate program effectiveness; (4) using physical space to establish cardiac clinics; and (5) using the communications department to develop patient education materials.

Potential challenges were quickly identified. The nature of the lattice network was not consistent throughout the country. For example, the Regional Centers of Excellence had varying degrees of administrative and human resource capacity, so a core package of interventions for the RHD program had to be substantially altered according to local circumstances. The coverage of the JCRC network was not consistent across the country—with some areas of high-density coverage and other areas with substantial gaps in coverage. The JCRC infrastructure was designed primarily to conduct research studies and was less suited to providing clinical care. Furthermore, because of persistent stigma, there was a chance that some HIV-uninfected patients with RHD would be uncomfortable seeking care at a site known to previously provide HIV care. Finally, RHD care requires occasional consultation with specialists, but the UHI physicians were few in number and concentrated geographically in the capital city of Kampala. New insights from both patients and providers were needed to figure out how to surmount these challenges.

### New insights from patients

In order to engage patients, our team conducted a series of focus group discussions (FGD) with patients who suffered from RHD. These FGD were conducted by trained social scientists with institutional review board approval and according to standard qualitative research methods. Our methods and results have been reported separately [23-25]. In these studies, patients provided the following key insights on how to improve RHD care in Uganda.

#### Penicillin adherence among patients with RHD.

Our initial FGDs were designed to elicit barriers and facilitators of penicillin adherence using a socioecological model of health to frame the discussion [23]. Lack of resources and injection site pain were personal barriers to penicillin adherence. Similarly, interpersonal barriers included lack of family/social support. Unskilled health care providers, penicillin stock-outs, and long wait times were systemic barriers. Patients suggested that interventions aimed at improving social support (e.g., home visits) and reducing barriers to getting to clinic (e.g., reimbursement for bus fare) would greatly facilitate adherence. While measures to reduce injection site pain were greatly appreciated, its fear was not typically the primary reason why patients missed their penicillin injections. These insights contrasted with our team’s preconceived notions about why patients may not be adherent. Finally, we also conducted a FGD among a small number of HIV-infected patients who also had RHD. We found that learning how to be adherent to daily antiretroviral therapy helped these patients understand the chronic nature of RHD and helped them to be more adherent to their penicillin injections. Perhaps these patients with HIV and RHD would be effective peer counselors for those who had trouble with adherence.

#### Primary prevention of RHD.

Because acute RF and RHD result from repeated streptococcal throat infections in children, we wanted to know about the health care practices of children and their caretakers. In FGDs, we discovered that children rarely reported having a sore throat, and that when they did, their parents first took them to a traditional healer for care [24]. If antibiotics were administered, they typically could only afford 1 or 2 doses. Children and adults also had very poor baseline awareness of RHD, but they were eager to learn more. Caregivers suggested that radio and television advertisements in the local language would be the most effective way to educate the public, as not all children attended school.

#### Maternal health issues for women with RHD.

A special population of patients with RHD are women of reproductive age. Because pregnancy puts increased demands on the heart, these women are at increased risk of decompensation and death [26]. We were curious to know whether women of reproductive age were being counseled on the risks of getting pregnant and whether they had adequate access to family planning services. Women in our FGD felt strongly that others would look down on them if they were unable to have children due to a heart condition and therefore saw pregnancy as a calculated risk [25]. Often, their reproductive decision making was controlled by male partners or in-laws, and few were aware of the birth defects caused by some of the medicines they were taking. These women felt that a clinic especially designed to take care of pregnant women with cardiovascular diseases would be beneficial.

### New insights from providers

Ugandan partners have been instrumental in designing the nature of our many programs, including the RHD treatment network. The insights from Ugandan providers came from ongoing conversations about program development rather than from formal FGD or qualitative research. Two main themes emerged from that conversations about how to improve cardiovascular care throughout the country.

#### Advanced training.

Until recently, advanced cardiovascular services offered at the UHI, such as complex congenital heart surgery, were dependent largely on visiting surgical teams from Europe and the United States. Yet, the need was much larger than the capacity this strategy provided. On the adult side, this was even more apparent, as a catheterization laboratory existed, but no Ugandan physician was capable of performing procedures independently. For RHD patients in particular, a large number of patients with rheumatic mitral stenosis could be treated with percutaneous balloon mitral valvuloplasty if the expertise was available locally. Ultimately, if enough experts were trained in Uganda, there would no longer be a need for visiting teams.

Similarly to the UHI, physicians who were trained in HIV/AIDS care at JCRC desired opportunities for advanced training. As more HIV providers had been trained across the country, the JCRC providers found that they were no longer as busy as they used to be in the early days of the epidemic. Perhaps their skills as chronic care providers could be repurposed to treat patients with RHD.

#### Access to technology.

Our Ugandan colleagues frequently commented that the main reason physicians were leaving the country to practice in the United States or Europe was not primarily economic (although this did play a role), but rather frustration with not being able to accurately diagnose and effectively manage disease in a resource-limited setting. Although cardiologists in Kampala had access to echocardiography, treadmill stress testing, and a cardiac catheterization laboratory, providers practicing in other parts of the country were often left without even a sphygmomanometer. Although ultrasonography and computed tomography scanners were occasionally available at regional centers, when they broke down it took months or even years for their repair if needed. Fortunately, over the last decade, technological innovation has led to high-quality ultrasonography being packaged into small handheld units. These units were less expensive and could be easily sent for repair if necessary. Thus, handheld ultrasonography might be used to help health care workers in remote regions of the country to accurately diagnose conditions such as RHD and empower them to do the work they were trained to do in medical school.

In the next section, we describe how our collaboration implemented these new ideas from patients and providers to deliver value—higher quality heart care at lower cost— for all Ugandans. The effort has begun with the RHD program rolled out over a lattice of HIV/AIDS infrastructure, but the vision is for a self-sustaining network providing care to patients with all sorts of heart diseases.

## DELIVERING VALUE

### The Regional Centers of Excellence model

**FIGURE 2.**
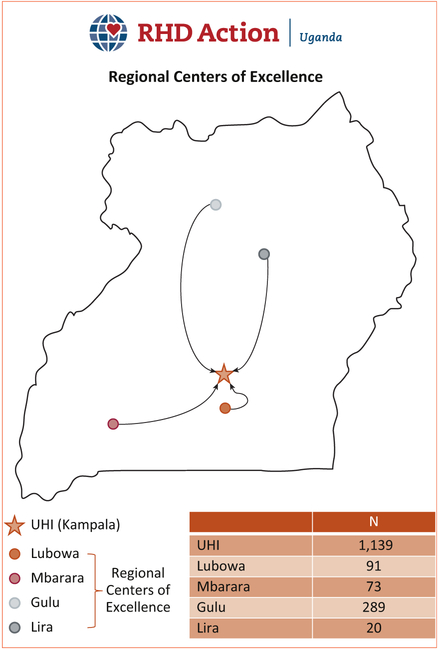
Regional Centers of Excellence for RHD Care in Uganda. The chart displays the total number of patients enrolled in the national registry per site as of January 2017. RHD, rheumatic heart disease; UHI, Uganda Heart Institute.

The core activity of RHD Action—Uganda was to create a network of Regional Centers of Excellence that could provide core RHD services closer to where patients lived. The program began in the region surrounding the capital city of Kampala, with the creation of a regional center at JCRC—Lubowa in the first year. Subsequently, the program expanded to Mbarara, Gulu, and Lira (Figure 2). Local conditions determined how HIV/AIDS infrastructure could be repurposed for RHD care. At Lubowa, for example, plentiful physical space allowed for the construction of a cardiac clinic. In Gulu, the JCRC facility was located 10 km outside of town, which was impractical for seeing large numbers of patients. Physician champions were not equally available at all locations. Similarly, administrative and support staff were more or less available at certain locations based on other projects that demanded their time and attention. Over the years, we have determined that a core team of 1 to 2 specialized nurses with at least 1 physician champion, along with an ultrasonography machine (or multiple handheld machines), and wireless mobile Internet access and airtime (for data management and communication) are required to operate a regional center. The collaboration is poised to take this model to other regional centers in future years, based on priorities of the collaborating partners and the Ministry of Health.

### A national RHD registry

**FIGURE 3.**
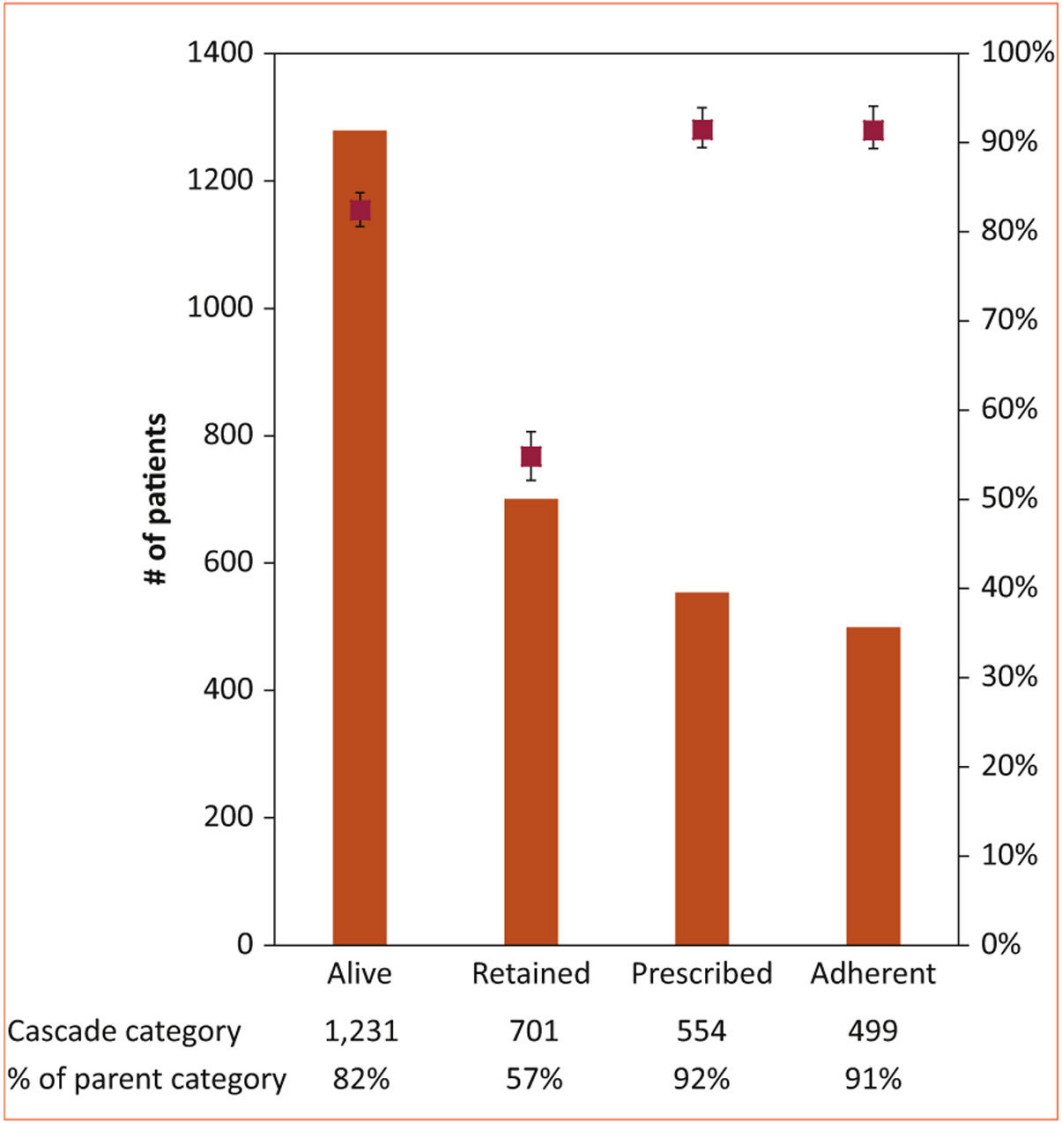
The RHD treatment cascade in Uganda. The **left** axis and **orange** bars indicate the number of patients in each outcome category of the treatment cascade, whereas the **right** axis and **red** points indicate the percentage of patients as a proportion of the parent (prior) category. Error bars reflect the 95% confidence interval. All patients in the registry were included to assess outcomes of alive and retained, but patients with borderline RHD were excluded from assessing the outcomes of prescribed and adherent. Used with permission from Longenecker et al.[27]RHD, rheumatic heart disease.

A key requirement of coordinated chronic care is the existence of a register to keep track of patients, schedule appointments, and track adherence to therapy. The collaboration decided to create a web-based RHD registry that could be easily accessible at each regional site. Thus, a national record was created that could be queried at any time for clinical or epidemiological purposes. The RHD Registry is approved by U.S. and Ugandan institutional review boards and all patients sign written informed consent to have their clinical information collected for epidemiological research. In addition, providers felt that it was important to develop metrics around how the system was functioning and whether specific interventions might work. Here again, we borrowed from HIV/AIDS models to create the RHD Treatment Cascade (Figure 3) [27]. We found that retention in care was the most significant barrier along the cascade, while rates of optimal adherence to benzathine penicillin G were high among those patients who were retained in care [27]. Our analysis also showed that distance to a local health center and access to a regional RHD center of excellence were associated with improved retention and adherence, suggesting that our efforts to decentralize care had significant impact on these care quality metrics [27].

### Penicillin

Penicillin adherence for patients with known RHD (i.e., secondary prevention) is the primary outcome measure targeted by RHD Action—Uganda. Our FGD with patients and data from the national registry taught us that interventions should be targeted toward retention in care [23]. Some regional centers experimented with transportation reimbursement for certain patients, whereas home visits from social workers were required in other cases. A peer-support group comprising patients with RHD has recently been formed that provides a program of peer-counseling for patients who are lost to follow-up or not retained in care. Because patients described frequent stock-outs of penicillin at local clinics, we began giving some patients a 3-month supply of penicillin to take home with them every time they came to see the doctor at the regional center.

### Maternal health

As mentioned previously, pregnant mothers with RHD and other forms of cardiovascular diseases are at high risk for decompensation and death [26]. In response to feedback from both patients and providers, we have been developing interventions to address the unique issues of this population. A physician leader at UHI is establishing a maternal cardiovascular care clinic that will provide coordinated consultative services for these high-risk patients. Additionally, in a project led by Children’s National Medical Center and Imaging the World (a nongovernmental organization that aims to bring accessible, affordable, and high-quality ultrasonography to remote and underserved communities), mothers are being screened for RHD with echocardiography at the time of prenatal ultrasonography in several locations around the country. As is the case throughout sub-Saharan Africa, there is a substantial overlap between the HIV/AIDS care lattice and maternal-child health lattice at the governmental and nongovernmental levels [28], providing opportunity to bring in additional partners with mutual interest in improving RHD care and heart care more broadly.

### Technology

The message from both patients and providers is that technology has the potential to revolutionize health care in Uganda, but a focus on value is critically important. One needs to look no further than the mobile phone to understand the profound improvements in standard of living that technology can provide. Handheld ultrasonography is such a technology that has the power to revolutionize heart care, especially in more rural areas of Uganda.

With colleagues from Children’s National Medical Center in Washington, DC, we are conducting a trial of handheld ultrasonography to improve diagnosis of heart failure symptoms at the Lira Regional Centre. Selected nonspecialist physicians, clinical officers, and nurses have learned to use the Vscan (General Electric Healthcare, Little Chalfont, United Kingdom) to diagnose RHD, dilated cardiomyopathy, hypertensive heart disease, pericardial effusion/tamponade, isolated right heart failure, and congenital heart diseases. These major categories of heart failure symptoms have dramatically different treatments. Prior to the introduction of ultrasonography, patients without a cardiac cause of their symptoms were often treated as having heart disease, whereas many with heart disease were often given a noncardiac diagnosis such as pneumonia. We hope that access to this simple diagnostic tool in the hands of nonspecialists will dramatically improve patient outcomes.

### Training

**FIGURE 4.**
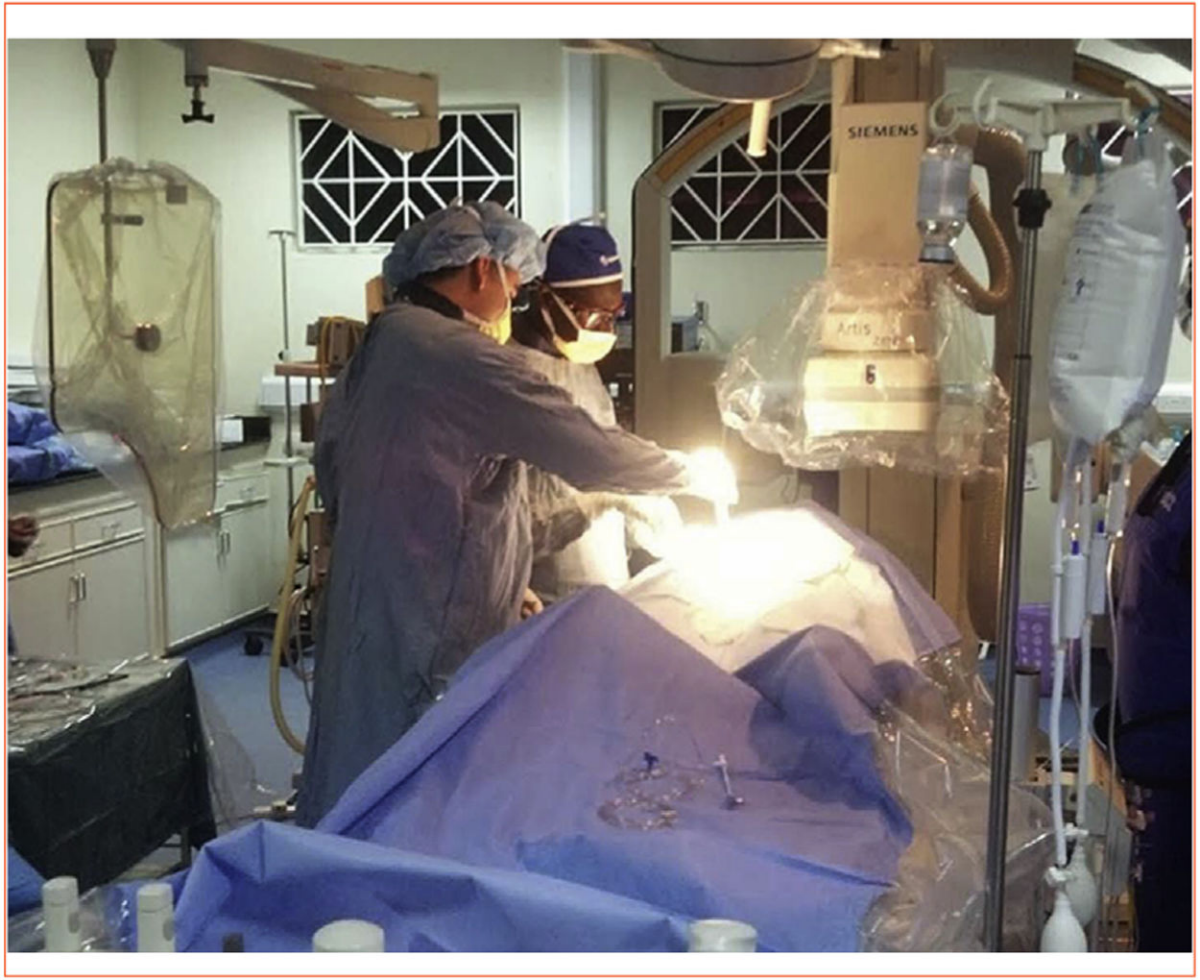
Dr. Marco A. Costa and Dr. Emmy Okello performing the first percutaneous mitral valvuloplasty for rheumatic mitral stenosis in August 2012.

Training has been a core principle of RHD Action from the very beginning. The Ugandan providers demanded it, and there was no other option to solve the profound human resource problem. We were fortunate to be able to provide targeted academic exchanges with Makerere faculty through Ohio’s Clinical Research Faculty Certificate program [29]. In this way, 2 UHI cardiologists were able to receive advanced experience in interventional cardiology techniques in exchange for teaching medical residents and students at UH/CWRU. Two additional UHI cardiologists trained in advanced heart failure in Cleveland, and an anesthesiologist is currently training in cardiac anesthesia and critical care. As evidenced by the large number of partners and diverse funding sources (Tables 1 and 2), the cardiovascular care development program in Uganda also builds on a lattice of relationships that have been forged because of mutual interests and backgrounds. Our relationship with colleagues in Brazil facilitated travel of Ugandan physicians to Belo Horizonte in Minas Gerais to learn percutaneous balloon mitral valvuloplasty for rheumatic mitral stenosis. The first percutaneous balloon mitral valvuloplasties were performed by the UH/CWRU visiting team in August 2012 (Figure 4). Four years later, in December 2016, the UHI team performed their first independent valvuloplasty and is now growing the volume of these procedures with the aim to become a center of excellence for mitral valvuloplasty in East Africa.

Nursing education in Uganda has not historically included research or higher level administrative skills, but several nurses requested to receive training and mentorship through working with us on special projects. By closely involving nurses in ongoing research projects, we hope to inspire young nurses to ask novel and interesting questions about how to advance health in their unique settings. Two examples are a nurse who directs a project on handheld ultrasound in Lira, and another nurse who is being mentored to transition into a nurse coordinator for the Gulu Regional Center.

## CONCLUSIONS

The Ugandan health care system is clearly under-resourced to provide high-quality heart care to all of its 36 million citizens; however, the Ugandan Ministry of Health has committed substantial resources to support UHI’s vision to decentralize its services and improve referral networks across the country over the next decade. The results of our initial success with the RHD Action pilot program illustrate how the government might leverage existing latticework (such as HIV/AIDS clinics or maternal-child health programs) to provide core NCD services and even more specialized forms of heart care. The UH/CWRU—Uganda partnership will remain critical to future scalability by continuing to provide research and training opportunities through an iterative and continuous cycle of program development. For some problems, we have been through the cycle 2 or 3 times; for others, the cycle is just beginning. Regardless, the future of heart care in Uganda is bright as long as the focus remains human-centered on patients and their providers.

## Figures and Tables

**TABLE 1.** Partners for the Ugandan cardiovascular care development program

Uganda	International
Uganda Ministry of Health	Children’s National Health System (Washington, DC, USA)
Uganda Heart Institute	Federal University of Minas Gerais (Belo Horizonte, Brazil)
Joint Clinical Research Center	World Heart Federation (Geneva, Switzerland)
Makerere University	RhEACH: Rheumatic Heart Disease Evidence-Advocacy-Communication-Hope (Perth, Australia; Cape Town, South Africa)
Mbarara University of Sciences & Technology	Pan-African Society of Cardiology (Cape Town, South Africa)
Gulu University	The Salam Centre for Cardiac Surgery (Khartoum, Sudan) Imaging the World (Charlotte, VT, USA)

**TABLE 2.** Funding sources for the Ugandan cardiovascular care development program.

Medtronic Global Health Foundation (Minneapolis, MN, USA)	RHD Action ($6 million; 2014 to 2019) RHD Action Uganda ($1.3 million; PI Longenecker 2013 to 2018)
NIH and Fogarty International Center (Bethesda, MD, USA)	Medical Education Partnership Initiative (MEPI-CVD; PI Sewankambo; $2.5 million) NURTURE: Research Training and Mentoring Program for Career Development of Faculty at Makerere University College of Health Sciences (PI Sewankambo; $4 million)
American Heart Association (Dallas, TX, USA)	Strategically Focused Research Network Grant ($3.7 million, PI Sable (CNHS); 2017 to 2021)
THRiVE Consortium (Kampala, Uganda, and Cambridge, UK)	THRiVE-2 Fellowship (PI Okello; $75,000)
Gift of Life and Rotary International (Evanston, IL, USA)	Through CNHS partners; congenital heart disease surgical program; Supplemental support of Gulu and Lira Regional Centers
General Electric Healthcare (Chicago, IL, USA)	Ultrasonography donation

CNHS, Children’s National Health System; MEPI-CVD, Medical Education Partnership Initiative Cardiovascular Disease linked award; NIH, National Institutes of Health; PI, principal investigator; RHD, rheumatic heart disease; THRiVE, Training Health Researchers into Vocational Excellence in East Africa.

## References

[1] GBD 2016 Disease and Injury Incidence and Prevalence Collaborators. Global, regional, and national incidence, prevalence, and years lived with disability for 328 diseases and injuries for 195 countries, 1990–2016: a systematic analysis for the Global Burden of Disease Study 2016. Lancet 2017;390:1211–59. 28919117 10.1016/S0140-6736(17)32154-2 PMC5605509

[2] Duffy M, Ojikutu B, Andrian S, Sohng E, Minior T, Hirschhorn LR. Non-communicable diseases and HIV care and treatment: models of integrated service delivery. Trop Med Int Health 2017;22:926–37. 28544500 10.1111/tmi.12901

[3] Barasa FA, Vedanthan R, Pastakia SD, Approaches to sustainable capacity building for cardiovascular disease care in Kenya. Cardiol Clin 2017;35:145–52. 27886785 10.1016/j.ccl.2016.08.014

[4] Cohen RL, Li Y, Giese R, Mancuso JD. An evaluation of the President’s Emergency Plan for AIDS Reliefeffect on health systems strengthening in sub-Saharan Africa. J Acquir Immune Defic Syndr 2013;62:471–9. 23254150 10.1097/QAI.0b013e3182816a86

[5] Lohman N, Hagopian A, Luboga SA, District health officer perceptions of PEPFAR’s influence on the health system in Uganda, 2005–2011. Int J Health Policy Manag 2016;6:83–95. 28812783 10.15171/ijhpm.2016.98 PMC5287933

[6] Paton NI, Kityo C, Hoppe A, , for the EARNEST Trial Team. Assessment of second-line antiretroviral regimens for HIV therapy in Africa. N Engl J Med 2014;371:234–47. 25014688 10.1056/NEJMoa1311274

[7] Abongomera G, Kiwuwa-Muyingo S, Revill P, , for the Lablite Project Team. Population level usage of health services, and HIV testing and care, prior to decentralization of antiretroviral therapy in Agago District in rural Northern Uganda. BMC Health Serv Res 2015;15:527. 26615587 10.1186/s12913-015-1194-4 PMC4662831

[8] Aliku TO, Lubega S, Namuyonga J, Pediatric cardiovascular care in Uganda: current status, challenges, and opportunities for the future. Ann Pediatr Cardiol 2017;10:50–7. 28163428 10.4103/0974-2069.197069 PMC5241845

[9] Longenecker CT, Okello E, Lwabi P, Costa MA, Simon DI, Salata RA. Management of rheumatic heart disease in Uganda: the emerging epidemic of non-AIDS comorbidity in resource-limited settings. J Acquir Immune Defic Syndr 2014;65:e79–80. 24442227 10.1097/QAI.0b013e3182a03eb9 PMC4423536

[10] Sika-Paotonu D, Beaton A, Raghu A, Steer A, Carapetis J. Acute rheumatic fever and rheumatic heart disease In: Ferretti JJ, Stevens DL, Fischetti VA, editors. Streptococcus pyogenes: Basic Biologyto Clinical Manifestations. Oklahoma City, OK: University of Oklahoma Health Sciences Center; 2016 p. 1–57. 28379675

[11] Watkins DA, Johnson CO, Colquhoun SM, Global, regional, and national burden of rheumatic heart disease, 1990–2015. N Engl J Med 2017;377:713–22. 28834488 10.1056/NEJMoa1603693

[12] Longenecker CT, Lwabi P, Kityo C, Leveraging existing HIV/AIDS infrastructure for rheumatic heart disease care in Uganda: a collaborative disease surveillance and management program. Glob Heart 2014;9:e55.

[13] Manyemba J, Mayosi BM. Penicillin for secondary prevention of rheumatic fever. Cochrane Database Syst Rev 2002;(3):CD002227. 12137650 10.1002/14651858.CD002227 PMC7017848

[14] Schwartz JI, Dunkle A, Akiteng AR, Towards reframing health service delivery in Uganda: the Uganda Initiative for Integrated Management of Non-Communicable Diseases. Glob Health Action 2015;8:26537. 25563451 10.3402/gha.v8.26537 PMC4292588

[15] Schwartz JI, Guwatudde D, Nugent R, Kiiza CM. Looking at non-communicable diseases in Uganda through a local lens: an analysis using locally derived data. Global Health 2014;10:77. 25406738 10.1186/s12992-014-0077-5 PMC4240853

[16] Odekunle FF, Odekunle RO. The impact of the US president’s emergency plan for AIDS relief (PEPFAR) HIV and AIDS program on the Nigerian health system. Pan Afr Med J 2016;25:143. 28292105 10.11604/pamj.2016.25.143.9987 PMC5326074

[17] Matheson GO, Pacione C, Shultz RK, Klugl M. Leveraging human-centered design in chronic disease prevention. Am J Prev Med 2015;48:472–9. 25700655 10.1016/j.amepre.2014.10.014

[18] Gupta N, Bukhman G. Leveraging the lessons learned from HIV/AIDS for coordinated chronic care delivery in resource-poor settings. Healthc (Amst) 2015;3:215–20. 26699346 10.1016/j.hjdsi.2015.09.006

[19] Rabkin M, Nishtar S. Scaling up chronic care systems: leveraging HIV programs to support noncommunicable disease services. J Acquir Immune Defic Syndr 2011;57(Suppl 2):S87–90. 21857304 10.1097/QAI.0b013e31821db92a

[20] Rabkin M, Kruk ME, El-Sadr WM. HIV, aging and continuity care: strengthening health systems to support services fornoncommunicable diseases in low-income countries. AIDS 2012;26(Suppl 1):S77–83. 22781180 10.1097/QAD.0b013e3283558430

[21] Gardner EM, McLees MP, Steiner JF, del Rio C, Burman WJ. The spectrum of engagement in HIV care and its relevance to test-and-treat strategies for prevention of HIV infection. Clin Infect Dis 2011;52:793–800. 21367734 10.1093/cid/ciq243 PMC3106261

[22] US Department of Health and Human Services. HIV Care Continuum. 2017 Available at: https://www.hiv.gov/federal-response/policies-issues/hiv-aids-care-continuum Accessed August 17, 2017.

[23] Huck DM, Nalubwama H, Longenecker CT, Frank SH, Okello E, Webel AR. A qualitative examination of secondary prophylaxis in rheumatic heart disease: factors influencing adherence to secondary prophylaxis in Uganda. Glob Heart 2015;10:63–69.e1. 25754568 10.1016/j.gheart.2014.10.001

[24] Moini B, Hanson JE, Webel A, Nalubwama H, Salata R, Longenecker C. Promoting primary prevention of rheumatic heart disease in Uganda: a qualitative study of group A streptococcal pharyngitis awareness in urban and rural communities. Glob Heart 2014;9:e256.

[25] Chang AY, Nabaale J, Nalubwama H, Characteristics and Motivations of Women of Reproductive Age in Uganda with Rheumatic Heart Disease: A Mixed Methods Study. Ann Glob Health 2017;83: 171–172.

[26] Sliwa K, Johnson MR, Zilla P, Roos-Hesselink JW. Management of valvular disease in pregnancy: a global perspective. Eur Heart J 2015; 36:1078–89. 25736252 10.1093/eurheartj/ehv050

[27] Longenecker CT, Morris SR, Aliku TO, Rheumatic heart disease treatment cascade in Uganda. Circ Cardiovasc Qual Outcomes 2017; 10:e004037. 29133472 10.1161/CIRCOUTCOMES.117.004037 PMC5728153

[28] Fowkes FJ, Draper BL, Hellard M, Stoove M. Achieving development goals for HIV, tuberculosis and malaria in sub-Saharan Africa through integrated antenatal care: barriers and challenges. BMC Med 2016;14:202. 27938369 10.1186/s12916-016-0753-9 PMC5151135

[29] LAWriter Ohio Laws and Rules. Ohio Revised Code 4731.293 Clinical Research Faculty Certificate. Available at: http://codes.ohio.gov/orc/4731.293 Accessed August 28, 2017.

